# Exercise benefits in metabolism on cardiovascular disease

**DOI:** 10.3389/fcvm.2026.1781202

**Published:** 2026-04-21

**Authors:** Yuxuan Guo, Qiang Zheng, Xianyue Wang, Ben Zhang, Tao Yan

**Affiliations:** Department of Cardiac Surgery, General Hospital of the Chinese People's Liberation Army Southern Theater Command, Guangzhou, China

**Keywords:** cardiorespiratory fitness, cardiovascular disease, energy metabolism, exercise, physical activity

## Abstract

Low physical activity (PA) is an independent predictor of cardiovascular disease. Exercise, as a non-pharmacological intervention for prevention and treatment, has been widely proven to have direct cardiovascular protective effects, including improving cardiopulmonary function and regulating cardiac energy metabolism through key molecular pathways such as the PI3 K/Akt, AMPK, mTOR, PPAR, and SIRT3 signaling, which optimize mitochondrial function and reduce oxidative stress. Simultaneously, it can indirectly promote cardiovascular health by reshaping a healthy gut microbiota and enhancing the body's overall metabolic environment. Therefore, examining the interactions between the cardiovascular system and various metabolic systems from a holistic perspective is both important and necessary to fully understand the multiple mechanisms by which exercise benefits cardiovascular health. This review will systematically describe the direct regulatory effects of exercise on cardiopulmonary function and cardiac energy metabolism. Building on this, it will explore how exercise influences the diversity and abundance of gut microbiota, the function of the gut barrier, and the mediation of key gut microbiota metabolites such as short-chain fatty acids. It will also examine the links between gut microbiota dysbiosis and major adverse cardiovascular events, along with the potential intervention mechanisms of exercise. From an integrated metabolism perspective, the review will comprehensively detail the pathways through which exercise provides cardiovascular protection by regulating the cardiovascular system, gut microbiota, and interactions among multiple metabolic systems. Finally, it adopts an analytical framework based on multi-omics integration and systems biology network analysis, thereby overcoming the limitations of traditional single-dimensional research and facilitating a more comprehensive, holistic understanding of the complex, multi-scale metabolic changes induced by exercise and the underlying cardiovascular-protective regulatory networks.

## 1 Introduction

Regular exercise is a fundamental aspect of promoting human health (1, 2), particularly benefiting the cardiovascular system (3–5). The American Heart Association considers cardiorespiratory fitness (CRF) as a vital clinical indicator (6), highlighting the importance of improving physiological parameters as a standard part of clinical care. In addition to enhancing CRF (6, 7), regular exercise plays a crucial role in preventing and fighting a broad spectrum of human diseases, including cardiometabolic disorders, while promoting longevity (6, 8, 9). Despite the implementation of important public health guidelines and related promotional efforts over the past thirty years, inadequate physical activity (PA) continues to be a key factor in high healthcare costs, a greater burden of chronic diseases, and significant loss of life years (8, 9). Cardiovascular disease (CVD) remains the leading cause of global mortality (10), with physical inactivity being a significant contributing factor (11, 12). As the heart is the most metabolically demanding organ in the body, disruptions in cardiac energy metabolism significantly contribute to various cardiovascular pathologies (13) and often worsen heart failure (14). The trend of physical inactivity starts during adolescence (ages 13–15), leading to an increased risk of cardiometabolic disorders (15). Accumulating 22–60 min of moderate-to-vigorous physical activity (MVPA) reduces the risk of excessive sedentary time (5, 16, 17). Regular physical activity is positively associated with a reduced risk of traditional CVD risk factors, such as hypertension (18), dyslipidemia (19), and diabetes.

Although substantial evidence confirms the cardiovascular benefits of exercise and shows the connection between sedentary behavior, cardiometabolic dysfunction, and CVDs, there are still critical gaps in existing knowledge. Existing studies often focus on individual indicators of exercise-induced metabolic changes or isolated cardiometabolic links, but they fail to systematically examine how overall energy metabolism changes caused by exercise work together to regulate cardiac function and influence the development of CVDs. At the same time, the specific mechanism by which exercise improves CVDs through regulating cardiac energy metabolism has not been fully clarified, especially regarding the interaction between systemic metabolic improvement and the heart's intrinsic metabolic remodeling. There is a paucity of integrated evidence on the relationship between exercise-induced metabolic changes and long-term CVD outcomes, especially in high-risk populations, such as adolescents with early exercise deprivation (15) or those with pre-existing cardiometabolic diseases. This limits the translation of basic research findings to clinical practice and public health strategies.

**Figure 1:**
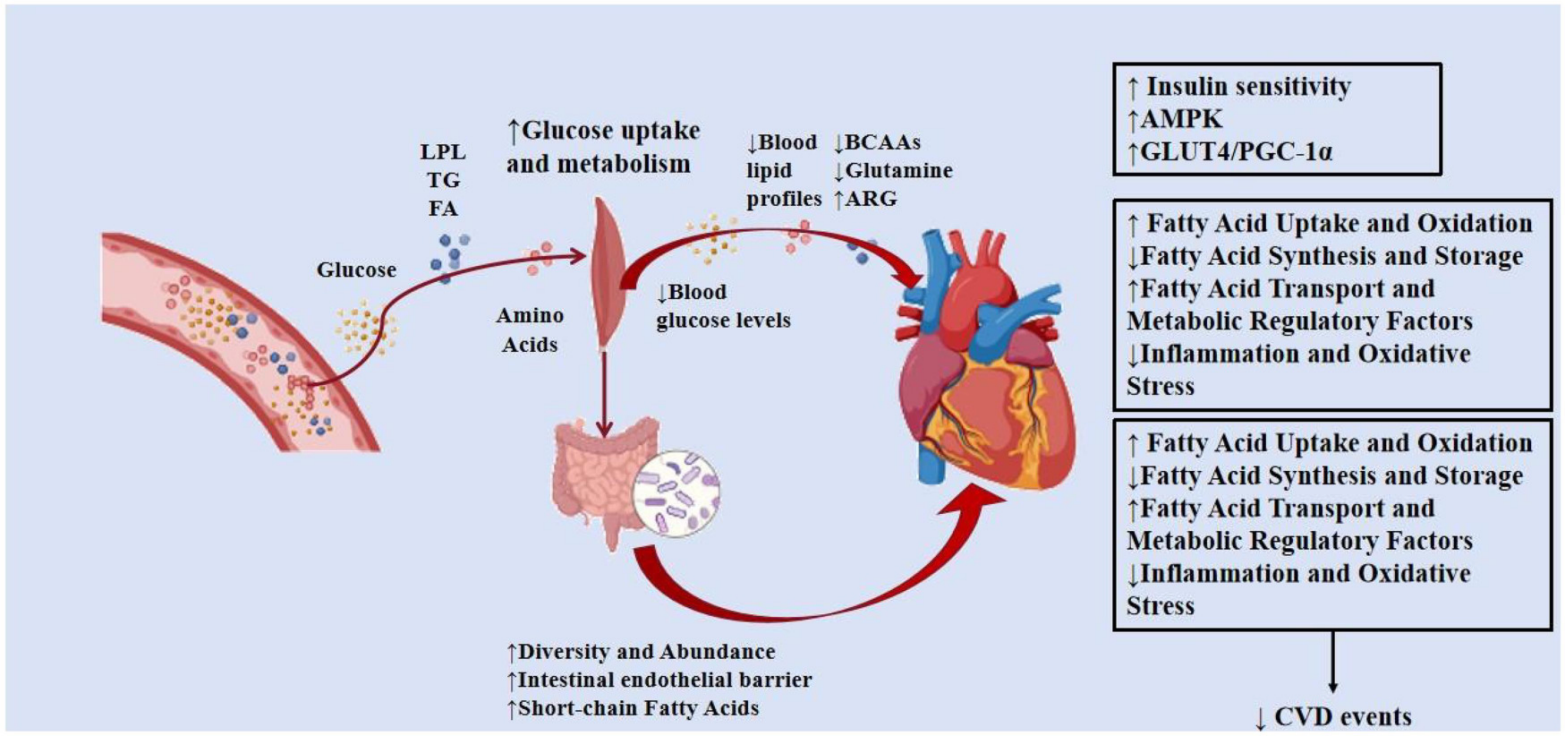
Graphical abstract.

To make up for the above research gaps, this review adopts an analytical framework based on the integration of multi-omics and systems biology network analysis, which can break through the inherent limitations of traditional single-dimensional research. Multi-omics technologies, including transcriptomics, metabolomics, proteomics, lipidomics, and microbiome, can comprehensively capture the molecular and metabolic changes induced by exercise across biological levels, while systems biology network analysis constructs and analyzes the interconnected regulatory network between key molecules, pathways, and tissues from a holistic perspective to identify the core regulatory hub and key mediators of exercise-induced cardiovascular protection. In this review, we provide an update on the effects of exercise-induced alterations in energy metabolism on the heart (Figure 1).

**Figure 2:**
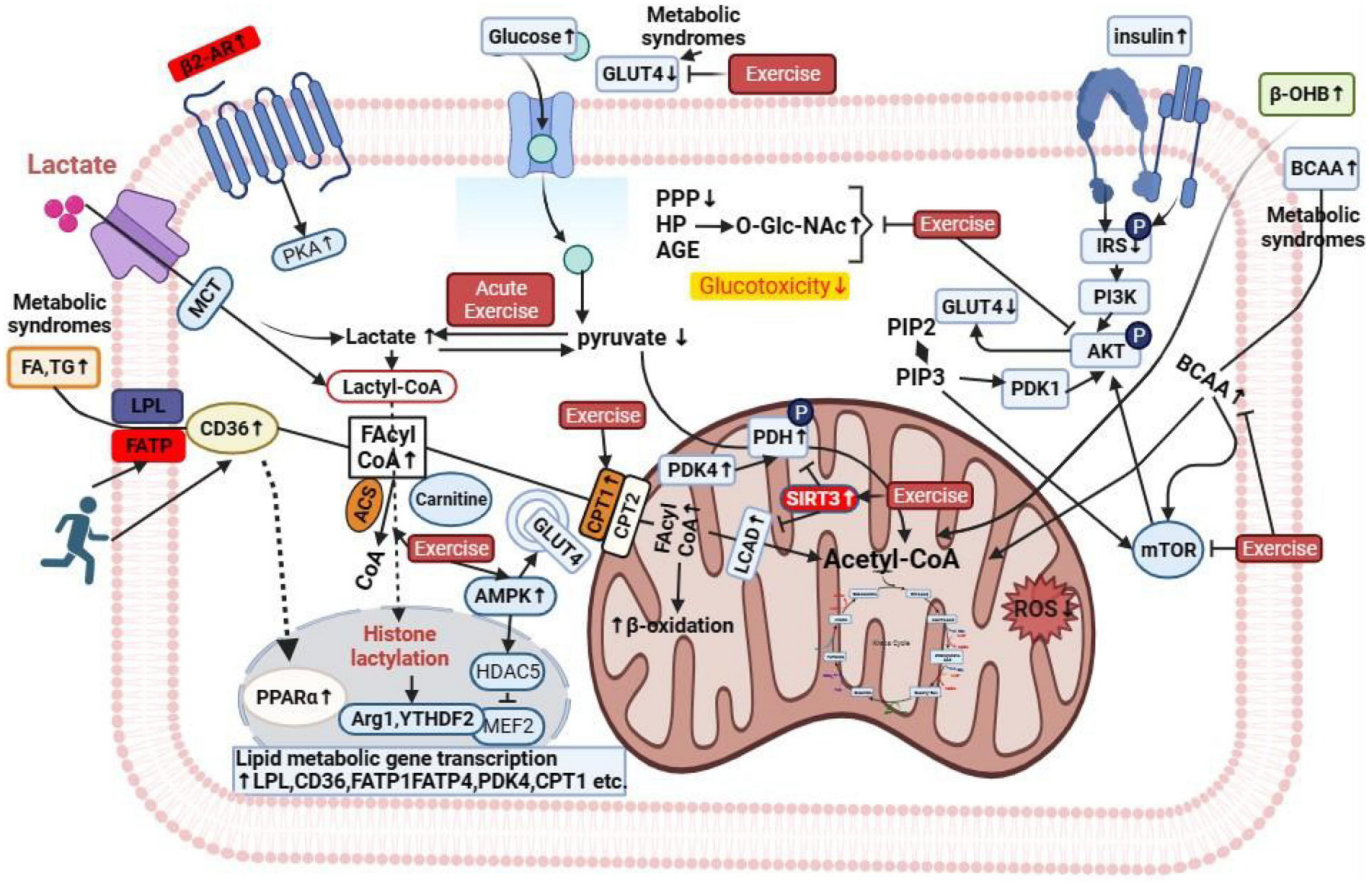
Main mechanisms explaining improvements in metabolism. Exercise decreased glucose uptake by down-regulating GLUT4 translocation. At the same time, it inhibits the pentose phosphate pathway (PPP) and hexosamine pathway (HP), reduces O-GlcNAc modification and the formation of advanced glycation end products (AGE), and directly reduces glucotoxicity. Acute exercise promotes the conversion of pyruvate to lactate, which is released through MCT or generates lactoyl-coa, providing a substrate for epigenetic regulation. Exercise activates AMPK and inhibits HDAC5, which in turn activates PPARα, up-regulates the key genes of lipid metabolism (such as LPL, CD36, FATP1/4, CPT1, etc.), and promotes fatty acid (FA) uptake and activation. Fatty acid acyl-coa (FAcyl-CoA) enters the mitochondria through CPT1/2 and generates acetyl-coa through β-oxidation, which provides energy for the tricarboxylic acid cycle and improves the efficiency of energy metabolism. Exercise activates the mitochondrial deacetylase SIRT3 to regulate the activity of PDH/PDK4 and precisely regulate pyruvate metabolism. At the same time, it enhances mitochondrial β-oxidation, reduces the generation of reactive oxygen species (ROS), and alleviates oxidative stress damage to cardiovascular system. In the state of metabolic syndrome, the level of branched-chain amino acids (BCAA) is increased, which activates the IRS-PI3K-AKT-mTOR pathway, inhibits GLUT4 transport, and aggravates insulin resistance. At the same time, the level of β-hydroxybutyric acid (β-OHB) is increased, which is related to metabolic disorders and cardiovascular risk. Exercise can improve abnormal insulin signaling by regulating mTOR and other nodes. FA, fatty acids; TG, Triglyceride; LPL, lipoprotein lipase; FATP, fatty acid transport proteins; MCT, monocarborxylat transporter; CD36, cluster of differentiation 36; BCAA, branched chain amino acid; ACS, fatty acyl-coa synthetase; CPT, carnitine palmitoyltransferase; LCAD, long-chain acyl-CoA dehydrogenase.

## 2 Exercise and energy metabolism

### 2.1 Exercise improves glucose metabolism

#### 2.1.1 Type 1 and 2 diabetes

Evidence from several studies has shown that exercise intensity is an important predictor of the magnitude of blood glucose reduction during PA in adolescents with type 1 diabetes (20, 21). Research indicates that adults with type 1 diabetes experience the greatest reduction in blood glucose levels during aerobic exercise (22). For those engaging in moderate-intensity exercise (MIE), the proportion of time spent within target blood glucose ranges (TIR) can decrease by up to 18%. Blood glucose levels did not decrease during high-intensity exercise (HIE) and resistance exercise (RE), which was attributed to elevated levels of catecholamines, cortisol, and growth hormone (23). Glycemic control after exercise, particularly in combination with exercise performance, has received little attention in the scientific literature. Recent studies have shown that an 8-week high-intensity interval training (HIIT) exercise in glucose-tolerant lean individuals, obese individuals, and type 2 diabetes (T2D) patients reduced ferritin levels in the serum, skeletal muscle, and liver (24), while increasing ferritin levels in white adipose tissue (WAT), thereby improving insulin sensitivity (25). Following intense exercise, the number of extracellular vesicles increases. These vesicles can transport glycolytic enzymes to recipient cells, potentially altering the rate of glycolysis in those cells (26). The specific mechanism is currently not clear, but it is unrelated to classical protein secretion. On the other hand, evidence from male muscle biopsies indicates that acute exercise-induced enhancement of muscle cell insulin sensitivity may stem from increased levels of glucose transporter type 4 (GLUT4) on the cell membrane (27), which contributes to preventing the onset of type 2 diabetes.

From animal models, during MIE, myocardial glucose oxidation increases by activating cardiac AMPK phosphorylation to increase GLUT4 (28), while it decreases during HIE (29, 30). GLUT4 knockout mice exhibit a significant reduction in *in vitro* glucose uptake during swimming exercise and muscle contraction periods. In a forced swimming exercise model with mice, it was observed that the exercise-induced upregulation of AMPKα (Thr172), total mTOR, ULK1 (Ser555), and LDHA (Ser196), while downregulating mTOR (Ser2448) and ULK1 (Ser757), promoting glycolysis (31). This helps the body resist chronic diseases such as T2D, CVD, and metabolic syndrome. Compared to the research on skeletal muscle, only HIIT mice showed an increase in myocardial citrate synthase activity (32). Long-term exercise increases the abundance of GLUT4 and hexokinase 2, promoting glucose uptake and increasing the levels of glucose-6-phosphate. Additionally, the increased mitochondrial volume and respiratory capacity resulting from long-term training enhance the capacity for energy metabolism, which may play a role in promoting insulin sensitivity (33). Studies in rodent models have shown that exercise increases muscle mitochondrial function (34–36) and insulin action by enhancing NAD+ (37) biosynthesis and overexpressing SIRT1-7 (38, 39).

Recent studies have shown that exercise effectively increases SIRT1 (40) expression in the heart tissue of diabetic rats, leading to increased angiogenesis (41) during vascular development (42), by inhibiting NF-KB, increasing NO production, and activating the MAPK/ERK and PI3K-Akt pathways (43). FGF21-SIRT3 is a novel protein involved in mitochondrial integrity and cardiac function in diabetic hearts, counteracting hyperacetylation (44) of SOD2 (45, 46), LCAD (45, 47), and several key components of mitochondrial complexes II and V (46), and contributing to the favorable effects of exercise on attenuating ROS/lipid accumulation and mitochondrial dysfunction in cardiomyocytes (48). It is well known that the heart can switch the fatty acid oxidation (FAO) mode to the glucose metabolism mode after myocardial infarction (MI), resulting in more ATP production per oxygen atom (49). And exercise modulation of energy metabolism is a potential new therapeutic option to rescue the myocardium. Micro-PET/CT scanning showed that myocardial glucose uptake increased after exercise (50). At the molecular level, exercise can activate AMPK, which in turn phosphorylates HDAC4, thereby increasing nuclear output of HDAC4. Thus, GLUT1 expression was increased by upregulating H3K9ac modification of the GLUT1 promoter and reducing MEF2a repression. Glycogen synthase kinase-3 beta (GSK-3β) can induce cell apoptosis by stimulating transcription factors. Another mechanism is that exercise activates the Akt-GSK-3β pathway. Phosphorylation of GSK-3β by Akt inhibits the opening of the mitochondrial permeability transition pore, ultimately increasing myocardial survival rate (51).

An *in vitro* study on rat cardiomyocytes indicates that glucose consumption and subsequent higher levels of aspartate as a nitrogen donor for nucleotide synthesis are necessary for promoting myocardial hypertrophy (52). In cultured 3T3-L1 adipocytes, insulin treatment rapidly induces translocation of the transferrin receptor to the plasma membrane (25). *In vitro* studies have demonstrated that exercise activates AMP-activated protein kinase (AMPK), which in turn positively regulates Rab5 activation. Rab5, a small GTPase, plays a key role in regulating GLUT4 translocation in both adipocytes and skeletal muscle cells. The TBC1D17–Rab5 axis governs the intracellular trafficking of GLUT1, GLUT4, and the transferrin receptor (53). This regulator*y* axis plays a pivotal role in mediating both glucose uptake and iron homeostasis in skeletal muscle during exercise. Evidence supporting the involvement of protein acetylation in the regulation of skeletal muscle glucose metabolism and insulin sensitivity *in vivo* is largely derived from studies employing gene knockout and transgenic mouse models (54). *In vitro* experiments have demonstrated that the I and II class histone deacetylase inhibitor, trichostatin A, increases glucose uptake and glycogen synthesis in C2C12 cells stimulated by insulin by enhancing the phosphorylation of INSR Tyr^1146^, AKT Ser^473^, and GSK-3β Ser^9^. Recent literature reports that overexpression of miR-19b-3p in primary mouse skeletal muscle cells augments insulin-stimulated glucose uptake through increased phosphorylation of Akt and TBC1D1 at serine residues (Figure 2) (55).

## 3 Exercise regulates fat metabolism

### 3.1 Obesity and metabolic syndromes

In 2022, Allard et al. conducted a study (STATEX trial) on the effects of cholesterol-lowering drugs on exercise performance and their impact on lipids and cardiometabolic parameters. As shown in Table 1, the study found consistent improvements in blood lipid status, obesity index, mitochondrial energy metabolism, and objective exercise ability after exercise (56). During exercise, fatty acids and ketones serve as key fuel sources for working muscles. As aerobic fitness capacity increases, there is a significant reduction in plasma levels of these metabolic substrates, including lipids, ketone bodies, fatty acid oxidation products, microbiome-derived metabolites, redox stress markers, and coagulation substrates (57). These reductions may be related to the improved efficiency of β-oxidation in skeletal muscle. Reducing plasma LDL-cholesterol levels through interventions that target intestinal cholesterol absorption, hepatic cholesterol synthesis, or hepatic LDL-cholesterol uptake could potentially reduce the prevalence of cardiovascular events in the general population (58). Recent meta-analyses have shown that exercise interventions, particularly aerobic interval exercise followed by resistance training, are effective in improving blood lipid profiles, including total and LDL-cholesterol, triglycerides, and HDL-cholesterol (especially the latter) (59).

**Table 1:** Impact of exercise training programs and cardiac rehabilitation on lipids and cardiometabolic parameters (taken from allard et al., 2022).

Content	Approximate % of test
Total cholesterol	−2%
HDL-C	−6%
LDL-C	−3%
Reductions in obesity indices	
BMI	−2%
% Fat	−2%
Improvements in exercise capacity	
maximal workload	20%
RT	11%
Proportion type I fibers	8.3%
Skeletal muscle mitochondrial function	
Citrate synthase activity	43%
ATP production capacity	25%

In a study involving middle-aged adults in the community, various exercise-induced brown adipose tissue-derived lipid factors and metabolites were found to be increased. These included 12,13-dihydroxy-9Z-octadecenoic acid (12,13-diHOME), which increases fatty acid uptake in skeletal muscle (60), as well as 13-hydroxyoctadecadienoic acid (13-HODE) (61), arachidonate, eicosapentaenoate/docosapentaenoate, 16-hydroxypalmitate hexadecanedioate, select sphingomyelins (C16:1 SM, C22:1 SM, C18:1 SM), medium- and long-chain acylcarnitines (C8:0, C10:0, C12:0, C14:0, C16:0, C18:0) (62). While some studies (63–65) did not observe significant differences in long-term weight loss and short-term outcomes between groups, others demonstrated that the exercise group achieved better long-term weight loss, with greater exercise leading to more weight loss (66). Magnetic resonance imaging (MRI) revealed that both types of exercise reduced epicardial adipose tissue mass in patients with abdominal obesity, while only resistance training reduced pericardial adipose tissue (67). The exact mechanism behind these effects remains unclear, but it may not be directly related to blood lipids, diabetes, or inflammatory markers (Figure 2) (68).

### 3.2 Coronary heart disease

 Large cohort studies have shown that exercise training has direct benefits for the heart and coronary vessels, including myocardial oxygen demand, endothelial function, autonomic nerve tone, coagulation and clotting factors, inflammatory markers, and the development of coronary collateral vessels (69). The reduction in its mortality may also be indirectly achieved through exercise by improving risk factors for atherosclerotic disease, such as blood lipids, smoking, and blood pressure (70). Exercise training has been reported to increase the vascular effects mediated by HDL cholesterol, such as nitric oxide (NO-) production and flow-mediated dilation (FMD), in patients with chronic heart failure (71, 72).

### 3.3 Metabolism pathway

Clinical investigations consistently demonstrate that exercise exerts profound regulatory effects on lipid metabolism, with mechanisms tightly linked to the modulation of key molecular targets, inflammatory responses, and metabolic crosstalk between tissues. In sedentary men undergoing 8 weeks of aerobic training, a 250% increase in maximal carnitine palmitoyltransferase 1 (CPT1) activity was observed in skeletal muscle, accompanied by upregulated mRNA expression of acyl-CoA synthetase (ACS), a key enzyme in fatty acid activation and oxidation (73). Notably, this training intervention also reduced CPT1 sensitivity to malonyl-CoA, thereby facilitating increased fatty acid entry into mitochondria for β-oxidation (74). HIIT stimulates lactate production, which in turn inhibits lipid synthesis via suppression of fatty acid synthase (FAS) activity (75). In human subjects, lactate, together with nonesterified fatty acids (NEFAs), serves as the primary fuel substrate for the heart during acute, moderate-intensity, and high-intensity exercise, highlighting a critical role of exercise-induced metabolic reprogramming in cardiac energy supply (30, 76, 77). Exercise-induced benefits on lipid metabolism are particularly prominent in populations at high risk of metabolic diseases, rather than healthy individuals (78). Specifically, exercise improves impaired metabolic activity by enhancing fatty acid uptake and activation in muscle cells, promoting fatty acid mitochondrial entry, and accelerating fatty acid β-oxidation, ultimately restoring the induced expression of adipokines such as adiponectin and spexin (79). These adipokines, whose levels decline with aging, play key roles in skeletal muscle insulin resistance and are closely associated with the pathogenesis of diabetes and other metabolic disorders (80). Beyond direct lipid metabolic regulation, exercise modulates age-related adipose-muscle crosstalk in metabolic diseases (81). It counteracts the senescence-associated secretory phenotype (SASP) and increases the release of myokines [e.g., myostatin, interleukin-6 (IL-6)], thereby promoting lipolysis and browning of white adipose tissue (82). Multiple cohort studies have shown that exercise-induced reductions in visceral fat are mediated by IL-6 (83, 84) and paracrine interleukin-11 (IL-11) (85) from bone tissue, further linking exercise-induced inflammation regulation with lipid metabolism homeostasis.

From animal models, consistent with human findings, rodent studies confirm that lactate, via binding to hydroxycarboxylic acid receptor 1 (HCAR-1) (86, 87), and NEFAs, are the major cardiac fuel substrates during exercise of varying intensities (30, 76, 77). Exercise persistently enhances fatty acid oxidation capacity in cardiomyocytes, a process partially mediated by peroxisome proliferator-activated receptor γ coactivator 1α (PGC-1α), a master regulator of mitochondrial biogenesis and fatty acid metabolism (88). Mechanistically, exercise activates AMPK, a central node in energy metabolic regulation, which promotes fatty acid catabolism and inhibits cholesterol synthesis (88). This activation induces phosphorylation of 3-hydroxy-3-methylglutaryl-CoA reductase (HMGR), the rate-limiting enzyme in cholesterol synthesis, and acetyl-CoA carboxylase (ACC), a key enzyme in fatty acid synthesis, thereby suppressing *de novo* lipid synthesis while enhancing fatty acid oxidation. Animal models have also confirmed exercise-induced modulation of lipid-related gene expression: exercise upregulates the expression of genes associated with fatty acid oxidation, including fatty acid translocase (FAT/CD36) and fatty acid oxidation-related enzymes (e.g., CPT1, ACSL1), while downregulating genes involved in fatty acid synthesis (e.g., FAS, ACC). Furthermore, exercise enhances the expression of cholesterol transporters (e.g., ABCA1, ABCG1) to promote reverse cholesterol transport and cholesterol efflux, and inhibits the expression of cholesterol synthesis-related genes (e.g., HMGCR). Additionally, exercise promotes weight maintenance through multiple mechanisms in animal models, including improved leptin sensitivity (89), enhanced sympathetic nerve tone, reduced hunger, increased satiety, promotion of dietary fat oxidation, and preservation of muscle mass (90).

Based on *in vitro* cell models, integrated transcriptomic and metabolomic analyses further complement the findings from single-pathway studies, providing a global perspective on exercise-induced lipid metabolic reprogramming. These multi-omics studies revealed a coordinated upregulation of genes related to fatty acid oxidation (such as CPT1 and ACSL1) and a concurrent downregulation of lipid synthesis genes (such as FAS and HMGCR) at the transcriptional level, accompanied by corresponding changes in the lipid metabolite profile (such as increased NEFAs and decreased triglycerides). Systems biology network analysis of these multi-omics data further revealed that AMPK acts as a central regulatory hub, linking fatty acid oxidation, cholesterol synthesis, and inflammatory response pathways, highlighting the synergistic interactions among these pathways that are critical for exercise-induced lipid homeostasis, insights that cannot be obtained by studying a single pathway alone. Consistent with human and animal studies, *in vitro* experiments demonstrate that exercise activates the AMPK pathway in macrophages to promote the biosynthesis of lipid catabolic mediators and regulate mitochondrial metabolism (88). Treatment of human adipocytes with oncostatin-M and 12,13-diHOME *in vitro* markedly enhances MAPK signaling and lipolysis, a mechanism that likely underlies exercise-induced anti-inflammatory effects and reduced CVD risk (Figure 2).

### 3.4 Circadian rhythm

From a clinical cohort, exercise responsiveness may exhibit temporal variability given that muscle strength, endurance, and athletic performance fluctuate diurnally and peak in the late afternoon (91). Afternoon exercise may have a greater impact on reducing circulating triglyceride levels and fasting blood glucose levels compared to morning exercise (92). Observational studies have yielded inconsistent conclusions regarding the impact of exercise timing on cardiometabolic outcomes (e.g., blood pressure, glycated hemoglobin, triglycerides): some studies support morning exercise, others favor afternoon/evening exercise, and additional investigations report no significant temporal differences (93). Experimental studies have similarly presented conflicting evidence, with one systematic review failing to identify robust support for time-dependent health benefits (94). Nevertheless, a meta-analysis demonstrated that afternoon/evening exercise could ameliorate glycemic control and reduce triglyceride levels, while another review indicated that morning exercise might be more conducive to weight loss (95). Collectively, the quality of evidence in this field remains suboptimal (93, 94), and future research should fully account for confounding factors, including medication use, postprandial status, sex, chronotype, and assessment time points, while also encompassing study populations spanning from children to older adults (96).

The cohort study also proved that exercise may modulate circadian health by exerting regulatory effects on sleep. PA, regardless of the timing of performance, generally improves sleep quality (97), though vigorous exercise within one hour before bedtime may reduce sleep efficiency in some individuals. Moderate-to-high intensity exercise can further strengthen circadian rhythmicity (98), and exercise timing may hold potential benefits for populations with circadian misalignment (e.g., shift workers, older adults, patients with circadian rhythm sleep disorders) (91). As a non-pharmacological intervention strategy, regular PA can promote circadian health by optimizing sleep quality, enhancing daytime alertness, and improving cardiometabolic outcomes.

For animal models, mesenchymal stem cells (MSCs) play a prominent role in mediating exercise-induced effects across multiple tissues and cell types. Exercise regulates pathways related to extracellular matrix remodeling and circadian rhythm in various tissues (99). In a healthy mouse model, exercise was found to induce an immediate increase in serum NEFAs and markers of mitochondrial proliferation specifically in inguinal adipose tissue, but these effects were observed only during early activity (100). After acute endurance exercise in mice, significant changes in the expression of core clock genes (including Bmal1, Clock, Cry1/2, Per1/2/3, Rev-Erbα, and Rorα) were observed (101). When exercising in the early morning during the day, without nighttime exercise, circadian rhythm-related transcriptional repressors (Ciart) and Per1 transcripts were induced and involved in the regulation of metabolism in skeletal muscle and liver (101). Transcriptomic and metabolomic analyses of mice showed that exercise during the early active phase immediately altered carbohydrate and adipose tissue metabolism, increased the expression of genes related to angiogenesis and glycolysis, as well as genes associated with fatty acid oxidation, branched-chain amino acid catabolism, and ketone metabolism (102). The above evidence suggests that exercise during the early active period may be more effective in treating metabolic disorders in both mice and humans.

*In vitro* experiments demonstrated time-dependent differences in the expression of β2 adrenergic receptors (ADRB2) in adipocytes. ADRB2 is a receptor involved in the regulation of lipolysis in adipose tissue. The time-dependent changes in ADRB2 expression suggest that the responsiveness of adipocytes to exercise-induced signals may vary depending on the timing of exercise (94).

## 4 Exercise improves amino acid metabolism

Recent studies have highlighted the impact of high-intensity aerobic training on amino acid metabolites and their role in improving cardiometabolic risk factors. After high-intensity aerobic training, there is an increase in amino acid metabolites in muscle tissue, which leads to a reduction in plasma concentrations of metabolites such as branched-chain amino acids (BCAA) (62), phenylalanine, GABA, aspartic acid, methylated arginine (ARG) metabolites (103), and methionine. This reduction in amino acid metabolites contributes to the improvement of cardiometabolic risk factors (104). In particular, acute exercise has been shown to increase blood ARG metabolism by 4%, enhancing vasodilation and decreasing the ornithine (105) /citrulline (106) ratio. A higher ornithine/citrulline ratio is associated with risk for metabolic syndrome (107). Moderate-intensity mixed aerobic and strength exercise increases ornithine content (57, 108, 109), potentially improving vascular function through the regulation of the growth hormone/insulin-like growth factor-1/insulin-like growth factor binding protein 3 complex in muscle tissue. Dimethylarginyl valerate (DMGV), a methylated metabolic derivative produced through the transamination of asymmetric dimethylarginine of arginine, is involved in nitric oxide signaling and vascular biology. DMGV is negatively correlated with personal responsiveness to exercise training (62, 110), and linearly positively correlated with the risk of coronary heart disease (111). It indicates that exercise may exert cardiovascular protective effects by lowering DMGV levels in the body, providing a potential molecular target and theoretical basis for exercise as a non-pharmacological intervention for the prevention and control of coronary heart disease.

Metabolomic studies in human populations have further revealed that low-to-moderate intensity exercise can elevate circulating 3-hydroxybutyrate levels (112). This effect is likely mediated by the positive regulatory role of long-term exercise training on gut microbiota (113), is not affected by gender (114), and is accompanied by characteristic changes in the cardiometabolic amino acid profile (e.g., serine, alanine, tyrosine, tryptophan, and BCAAs). Mechanistically, exercise induces the upregulation of peroxisome proliferator-activated receptor γ coactivator 1α (PGC1α) expression in human skeletal muscle, which in turn activates BCAA metabolism and promotes their utilization as an energy source via the tricarboxylic acid (TCA) cycle (115, 116).

Exercise also modulates the tryptophan-kynurenine (Kyn) pathway in humans: acute endurance exercise exerts a more significant inhibitory effect on Kyn pathway activity compared with resistance exercise (117), and the reduction in circulating kynurenine levels is correlated with decreased fasting blood glucose, glycated hemoglobin, cholesterol, and triglyceride levels (118). This regulatory effect of exercise on the tryptophan metabolic pathway is achieved by modulating the expression of two key enzymes: indoleamine 2,3-dioxygenase (IDO) and tryptophan 2,3-dioxygenase 2 (TDO2), which respectively mediate the initial and rate-limiting steps of the Kyn pathway. Additionally, exercise stimulates the production of N-lactoyl-phenylalanine (Lac-Phe), a blood-borne signaling metabolite that inhibits feeding behavior and alleviates obesity in humans.

From animal models, the metabolomics of mice showed that exercise significantly increased the levels of muscle acylcarnitine, BCAAs, and β-hydroxybutyric acid (BHBA), while accompanied by an increase in the expression of genes involved in fatty acid oxidation, BCAA catabolism, and ketogenesis (102). The lactic acid produced during exercise enters the liver cells and promotes the production of carboxylesterase. The lactic acid produced during exercise enters various types of cells and promotes the production of carboxylesterases. For example, liver cells secrete CES2A and CES2C, which have anti-obesity and anti-fatty degeneration effects (119). Skeletal muscles secrete lactyl phenylalanine, which reduces food intake in mice, alleviates obesity and weight gain, and improves glucose homeostasis (120).

*In vitro* cellular experiments have elaborated the detailed molecular mechanisms underlying exercise-mediated amino acid metabolism and angiogenesis, particularly the regulatory role of key transcription factors and signaling molecules in endothelial and muscle cells. In endothelial cells, activating transcription factor 3/4 (ATF3/4) directly regulates the expression of genes involved in amino acid uptake and metabolism; supplementation with nonessential amino acids and overexpression of ATF4 in cultured endothelial cells can significantly promote leukocyte endothelial cell proliferation. Conversely, the deletion of ATF4 in skeletal muscle cells impairs exercise-induced angiogenesis in *in vitro* cell models (121), highlighting the indispensable role of muscle cell amino acid metabolism in exercise-mediated vascular regeneration. Vascular endothelial growth factor (VEGF) orchestrates the adaptive response of cells to oxygen and nutrient deficiency during exercise, and *in vitro* studies have identified a partial mechanism for this effect: sulfur-containing amino acids can induce VEGF expression in cultured endothelial cells, which in turn mediates functional vascular regeneration in *in vivo* models (122). Furthermore, *in vitro* cellular assays have confirmed the synthetic source of Lac-Phe. CNDP2(+) cells (including immune cells and epithelial cells) possess the enzymatic capacity to synthesize Lac-Phe (120) from lactic acid and phenylalanine, which clarifies the cell-specific biosynthetic pathway of this key exercise-induced metabolite and provides a cellular basis for understanding its systemic metabolic effects.

The specific mechanisms by which different exercise modes improve CVD risk outcomes through the regulation of amino acid metabolism are not yet clear. This is even though multiple (105, 123–125) studies have shown evidence that different types of acute exercise reduce plasma BCAA in healthy lean and overweight individuals. A meta-analysis (126) that included acute exercise, 10 weeks of endurance exercise training (127), and 8 weeks of high-intensity interval training showed that patients with type 2 diabetes had significantly lower insulin sensitivity than obese and lean individuals, significantly higher plasma BCAAs levels, and reduced insulin ability to lower plasma BCAAs. Although exercise training significantly improved insulin sensitivity in all study populations, neither acute nor long-term exercise significantly improved plasma BCAAs levels nor the ability of insulin to regulate BCAAs. In addition, studies on the mechanism of BCAA metabolism showed that skeletal muscle was the dominant regulator of fasting plasma BCAAs levels, while liver was not involved in the regulation process of fasting plasma BCAAs, and reducing fasting plasma BCAAs concentration alone was not sufficient to achieve improvement of insulin sensitivity. No single tissue (skeletal muscle or liver) could explain the increased insulin sensitivity after pharmacological activation of BCKDH, suggesting that the synergistic regulation of BCAA metabolism by multiple tissues may be the key to improving insulin sensitivity and reducing cardiometabolic risk (128). It also clarified the core direction for the subsequent research on the metabolic regulation mechanism of BCAA (Figure 2).

## 5 Effects of exercise on the gut

### 5.1 Diversity and abundance

Clinical observations have demonstrated that, compared with sedentary individuals, professional athletes possess a gut microbiome with higher diversity and enhanced metabolic capacity relevant to cardiovascular health. In clinical training intervention studies, a significant increase in the abundance of butyrate-producing bacteria in the gut has been confirmed; this finding is further supported by complementary animal experiments (129, 130). Colonocytes serve as a major source of ketone bodies, specifically acetoacetate and 3-hydroxybutyrate, which can freely diffuse into the systemic circulation. Both clinical and animal experimental evidence have shown that exercise training can dynamically regulate the circulating levels of these ketone bodies, though the exact regulatory mechanism remains to be fully elucidated.

### 5.2 Intestinal barrier

Only moderate-to-vigorous intensity exercise more than 3 times per week for more than 8 weeks was consistently associated with changes in bacterial community and community structure (131). In this analysis, the health status of the investigated cohort led to variability and potential confounding of the results, and the definition of “health” was rather loose, covering individuals without clear criteria for excluding chronic diseases. At the same time, exercise can inhibit the growth of harmful bacteria related to the pathogenesis of cardiometabolic diseases, such as reducing the relative abundance of Desulfurivibrio (which produces hydrogen sulfide and damages the intestinal barrier) and Collinsella (which promotes inflammation and metabolic disorders), thereby improving the imbalance of intestinal flora (131).

### 5.3 Short-chain fatty acids

 Selective enrichment of short-chain fatty acids-producing bacteria and inhibition of the proliferation of harmful bacteria are the core of exercise regulation on intestinal flora community structure (131). Clinical studies have shown that exercise can induce significant changes in the genes of most Bacteroides and Clostridium species involved in the synthesis of SCFAs. In pre-diabetic individuals without drug treatment, the exercise-sensitive gut microbiota has a stronger ability to produce SCFAs and metabolic BCAAs (132). The post-race sample analysis of marathon runners showed that the abundance of Veillonellain their feces increased significantly (133), and Veillonella metabolized lactic acid to SCFAs through the methylmalonyl-coa pathway. This genus is closely related to the improvement of exercise endurance and the remodeling of cardiac energy metabolism. SCFAs-producing bacteria mainly include Firmicutes (such as Clostridium, Cecococcus, Faecalibacterium prausnitzii), Bacteroidetes, and Actinobacteria (such as Bifidobacterium). The abundance of these bacteria is directly related to the ability of SCFAs. High-intensity exercise (112, 131) has a more significant enrichment effect on SCFAs-producing bacteria. Emerging evidence (134) suggests that the distribution and abundance of SCFAs influence cardiometabolic health even more than the overall composition of the microbiota. Exercise-induced increase of SCFAs can significantly improve insulin resistance, reduce body fat accumulation, reduce serum levels of TNF-α, IL-6, hsCRP, and other pro-inflammatory factors, improve vascular endothelial function, regulate blood lipid and blood pressure homeostasis, and the above effects are significantly correlated with the concentration of SCFAs (135). From animal experiments, fecal samples from sedentary and trained animals transferred to germ-free mice showed increased levels of AMPK and insulin-like growth factor-1 in the skeletal muscle of recipient mice, along with improvements in systemic glucose and insulin sensitivity (131, 136).

### 5.4 CVDs

Transgenic sequencing of 8,973 patients with atherosclerosis (AS) found that the abundance of streptococci and Vibrio bacteria in the gut was associated with subclinical coronary atherosclerosis (137). A six-week resistance training program increases the relative abundance of Ruminococcus in obese individuals, lowers diastolic blood pressure, and improves cardiovascular health (138). Patients with ST-segment elevation myocardial infarction (STEMI) who exhibit elevated serum levels of gut-derived metabolites such as phenylacetylglutamine (PAGln), deoxycholic acid (DCA), trimethyllysine (TML), and trimethylamine-N-oxide (TMAO) face a higher risk of major adverse cardiovascular events (MACEs) (139). Bacteroides thetaiotaomicron can utilize host mucus glycosides to enhance the mucosal barrier and immune system, and is negatively correlated with systolic blood pressure (140). Compared with daytime exercise, nocturnal exercise in mice significantly reduced the size of As lesions in the aortic root, significantly reduced type III lesions (foam cells extending into the middle layer and lesions showing a fibrotic cap), promoted the enrichment of Firmicutes, and increased cell cyst-producing bacteria (141). Combined strength and endurance training for 12 weeks can increase the relative abundance of Roseburia, Prevotella, Clostridium, and Faecalibacterium, and reduce the relative abundance of Streptococcus and Clostridium (142), indicating that combined exercise can reduce the risk of AS in obese children. Twelve weeks of moderate intensity swimming exercise in mice attenuated cholesterol-driven plaque formation and main vessel thickening, and the relative abundance of Lactococcus was significantly reduced, suggesting that exercise may play a role in CVDs by reducing the abundance of Lactococcus as a potential pathogen (143).

In preclinical studies, chronic moderate-intensity exercise has demonstrated consistent cardioprotective effects mediated, at least in part, through modulation of the gut microbiota. 12 weeks of moderate-intensity treadmill exercise in spontaneously hypertensive rats (SHRs) induced a sustained reduction in systolic blood pressure, which correlated with increased microbial alpha diversity, enhanced taxonomic richness, enrichment of beneficial bacterial taxa (e.g., members of the genus Bifidobacterium and Lactobacillus), and resilience of these exercise-induced microbial adaptations following a 4-week detraining period (144). Similarly, 4 weeks of aerobic exercise in hypertensive (HTN) rats significantly increased the relative abundance of Heterofermentative bacteria while decreasing that of Aggregatibacter and Sutterella, shifts associated with attenuated systemic inflammation and reduced CVD risk (145). Plasma Lac-Phe is a top metabolite in exercise mice. Lac-Phe, after its CNDP2 mediated synthesis, mediates metformin mainly by inhibiting mitochondrial action in intestinal epithelial cells, thereby regulating appetite and body weight (146). Injection of Lac-Phe into high-fat feeding mouse model can inhibit central feeding behavior and reduce food intake, alleviate visceral fat accumulation and metabolic syndrome (120), promote Browning of white adipose tissue and enhance whole body energy expenditure, thereby reducing the cardiac load caused by obesity and hyperlipidemia (120). In murine models of MI, 8 weeks of endurance exercise elevated the relative abundance of Alistipes and Ruminococcus, and reduced that of Lachnospiraceae_UCG-001, collectively contributing to improved post-infarct cardiac function. It is worth noting that fecal microbiota transplantation (FMT) from mice with exercise-induced MI can promote more significant functional recovery. Compared with FMT from mice with sedentary MI, it is characterized by an increase in left ventricular ejection fraction and a reduction in fibrosis (106). Furthermore, 4 weeks of moderate-intensity exercise in MI mice significantly increased the abundance of several commensal and potentially beneficial genera, including Ruminococcus, Prevotella, Akkermansia, Butyricimonas, Erysipelotrichaceae, and Roseburia. These microbial alterations are closely associated with improvements in hemodynamic parameters. Exercise significantly increased cardiac output and stroke volume in MI mice, while concurrently enriching gut microbiota diversity and functionality (147). Mechanistically, Prevotella abundance has been inversely correlated with left ventricular ejection fraction in experimental models. Akkermansia muciniphila exerts well-documented anti-inflammatory effects in murine systems, and Butyricicoccus shows robust associations with improved glycemic control in insulin-resistant subjects (148, 149). Collectively, these findings support a microbiota-dependent pathway through which structured exercise confers cardiovascular protection.

## 6 Conclusion

Regular PA and exercise have been shown to have significant benefits in preventing CVDs (Figure 1). These effects are dose-dependent, meaning that the more regular and intense the exercise, the greater the benefits. This review systematically synthesizes the multi-dimensional mechanisms by which exercise exerts cardiovascular protection through the regulation of systemic energy metabolism, encompassing glucose, lipid, and amino acid metabolic remodeling, as well as indirect modulation via gut microbiota reshaping and circadian rhythm regulation. We clarify that exercise-mediated cardioprotection is not dependent on a single pathway or organ system, but rather relies on the synergistic cross-talk and integrated regulation of the cardiovascular system with peripheral metabolic tissues, gut microbiota, and circadian clock networks, with core molecular hubs including AMPK, SIRT1/3, PPARα, and GLUT4 serving as key mediators of these regulatory effects (Table 2).

**Table 2:** Key Molecular Mechanisms Related to Metabolism and Their Effects on Cardiovascular Health

Key Molecules/Pathways	Regulatory Effects	Inter-pathway Interactions	Protective Effects on Cardiovascular Health
AMPK Signaling Pathway	1. Promotes the translocation of GLUT4 to the cell membrane in cardiomyocytes and skeletal muscle cells, accelerating glucose uptake; 2. Phosphorylates HDAC4, upregulates H3K9ac modification of the GLUT1 promoter, relieves MEF2a inhibition, and increases GLUT1 expression; 3. Inhibits the opening of mitochondrial permeability transition pores, reduces cardiomyocyte apoptosis, and improves cardiomyocyte survival rate.	1. Interaction with GLUT4/GLUT1 regulation: AMPK is a key upstream regulatory factor of both; meanwhile, increased glucose uptake mediated by GLUT4/GLUT1 can feedback-activate AMPK, forming a positive regulatory loop; 2. Interaction with SIRT family: AMPK phosphorylates and activates SIRT1, and SIRT1 deacetylates and enhances AMPK activity, forming a synergistic axis; 3. Interaction with the Akt-GSK-3β pathway: AMPK activates PI3 K/Akt to indirectly regulate GSK3β, and Akt phosphorylates downstream substrates of AMPK to exert synergistic effects.	Optimizes myocardial energy supply and inhibits myocardial apoptosis; synergistically improves vascular endothelial function and inhibits cardiovascular inflammation; maintains glucose homeostasis and reduces glucotoxicity.
GLUT4/GLUT1 Regulation	1. GLUT4: As a key carrier for glucose uptake, its expression and translocation are regulated by the AMPK and Akt-GSK3β pathways, mediating rapid glucose uptake after exercise; 2. GLUT1: Mediates basal glucose transport, and its expression is increased through exercise-induced epigenetic modification (HDAC4 regulation) to supplement energy for cardiomyocytes and vascular cells.	1. Interaction with Akt-GSK-3β pathway: Akt promotes the fusion of GLUT4 vesicles with the cell membrane; inhibition of GSK-3β phosphorylation reduces its negative regulation on GLUT4, forming a dual-pathway synergistic regulation with AMPK; 2. Interaction with SIRT3: Increased glucose uptake optimizes mitochondrial energy supply, indirectly enhancing SIRT3 activity; SIRT3 improves mitochondrial function to feedback support GLUT4/GLUT1 translocation, forming a regulatory loop.	Improves insulin resistance and reduces hyperglycemia-related vascular endothelial injury; supplements energy for cardiomyocytes and vascular cells, alleviating myocardial ischemia and hypoxia; delays the progression of atherosclerosis.
SIRT Family Regulation	1. SIRT1: Increases expression in cardiac tissue, inhibits NF-*κ*B, promotes NO production, activates MAPK/ERK and PI3K-Akt pathways, and enhances angiogenesis and vascular endothelial function; 2. FGF21-SIRT3 axis: SIRT3 deacetylates SOD2, LCAD, etc., optimizes mitochondrial function, reduces ROS accumulation and lipid deposition, and alleviates diabetic cardiomyopathy.	1. Intrafamilial synergy: SIRT1 activates PI3K-Akt to promote FGF21 expression, which in turn activates SIRT3, forming a “SIRT1→FGF21→SIRT3” regulatory chain; 2. Interaction with AMPK: Bidirectional synergistic regulation to jointly enhance glucose metabolism and mitochondrial function; 3. Interaction with the Akt-GSK-3β pathway: SIRT1 deacetylation enhances Akt activity, and Akt phosphorylation feedback promotes SIRT1 expression, forming positive regulation.	Improves vascular endothelial function and promotes angiogenesis; reduces cardiovascular inflammation and ROS-induced injury; optimizes myocardial mitochondrial function, alleviates myocardial remodeling, and reduces the incidence of heart failure.
Akt-GSK-3β Pathway	1. Activates Akt and phosphorylates GSK-3β, inhibiting GSK-3β-induced cardiomyocyte apoptosis; 2. Promotes glycogen synthesis, reducing vascular endothelial injury caused by glucose accumulation; 3. Synergistically regulates GLUT4 translocation to enhance glucose uptake.	1. Interaction with AMPK and GLUT4/GLUT1: Forms dual-pathway synergistic regulation of GLUT4 with AMPK; AMPK directly activates Akt to enhance its anti-apoptotic function; 2. Interaction with auxiliary mechanisms: Glycolytic enzymes transported by extracellular vesicles and miR-19b-3p can both activate Akt phosphorylation, indirectly regulating GSK-3β to exert synergistic effects.	Inhibits cardiomyocyte apoptosis and alleviates myocardial remodeling; reduces vascular injury related to glucose accumulation; synergistically improves insulin resistance and reduces the risk of cardiovascular disease progression.
Other Auxiliary Mechanisms	1. Extracellular vesicles: Transport glycolytic enzymes to regulate the glycolytic rate of recipient cells; 2. miR-19b-3p: Increases serine phosphorylation of Akt and TBC1D1, enhancing insulin-stimulated glucose uptake; 3. Histone deacetylase inhibitors: Enhance phosphorylation of INSR, AKT, and GSK-3β, improving glucose uptake and glycogen synthesis.	Do not exert effects independently; mainly regulate the activity of core pathways (Akt-GSK-3β, AMPK) to enhance their regulatory effects and amplify exercise-induced improvements in glucose metabolism and cardiovascular protection.	Assist core pathways in exerting effects and amplify the regulatory effect of glucose metabolism; synergistically improve insulin sensitivity, reduce cardiovascular injury, and assist in lowering the risk of cardiovascular diseases.

The core strength of this review is the adoption of an analytical framework integrating multi-omics and systems biology, which breaks through the inherent limitations of traditional single-dimensional studies and achieves a comprehensive and holistic interpretation of exercise-induced metabolic changes. Multi-omics technology captures the dynamic multi-level molecular and metabolic changes caused by exercise at the genome, transcriptome, proteome, and metabolome levels. Systems biology network analysis further constructs and annotates the interconnected regulatory networks among key molecules, pathways, and tissues. This integrative approach not only identifies the core regulatory nodes and causal mechanistic links in exercise-mediated metabolic remodeling but also reveals the complex interplay among cardiac energy metabolism, gut microbiota-derived metabolites, and circadian metabolic signals, thus providing systematic insight into the multi-scale and multi-system nature of exercise cardioprotection. This is beyond the reach of isolated pathways or single omics studies.

Based on the research evidence collected in this review, combined with the needs of clinical practice and public health prevention and control, the following operational recommendations were provided for clinicians and policymakers. According to the European Society of Preventive Cardiology, it is recommended that adults perform 150–300 min of moderate-intensity or 75–150 min of vigorous-intensity aerobic exercise per week. Resistance training of moderate or higher intensity was required for more than 2 days, and the above exercise prescriptions followed the FITT principles (i.e., frequency, intensity, time, and type). For patients with type 2 diabetes, moderate-intensity aerobic exercise (such as fast walking, jogging, swimming) combined with resistance exercise (such as dumbbell training, elastic band training) 2–3 times a week, 30–45 min each time, and a total of more than 150 min per week (150). At the same time, metabolic indicators such as fasting blood glucose and glycosylated hemoglobin are monitored, and the exercise intensity is dynamically adjusted according to the changes in indicators. For obese people with metabolic syndrome, moderate intensity and long-term aerobic exercise should be given priority, combined with low-intensity resistance training, focusing on controlling body fat rate and improving insulin resistance, thereby reducing the risk of CVD. For healthy people and those at high risk of CVD, it is recommended to promote the “normal and low threshold” exercise mode, encourage 30 min of moderate-intensity exercise per day, reduce sedentary behavior, improve core metabolic indicators through exercise, and achieve the primary prevention of CVD.

Future research should focus on the feasibility of personalized exercise prescription and the application value of digital health technology, fill the existing literature gap, and improve the theoretical system of exercise metabolic regulation and cardiovascular protection. In terms of personalized exercise prescription, it is recommended to combine individual metabolic characteristics (such as basal metabolic rate, metabolic marker levels), intestinal microecological composition, CVD risk level, age, exercise tolerance, and other multi-dimensional indicators to build a personalized exercise prescription evaluation model. Machine learning, big data analysis, and other technologies can accurately match the type, intensity, and duration of exercise. To implement a “one person, one party” exercise intervention model, and to carry out prospective intervention studies to verify the effectiveness of personalized exercise prescription in improving metabolic disorders and reducing the risk of CVD. In terms of the application of digital health technology, the development and popularization of wearable devices (such as smart wristbands, sports watches) and portable metabolic monitoring instruments have been promoted to achieve real-time dynamic monitoring of metabolic indicators such as heart rate, blood oxygen, blood glucose, and fat burning efficiency during exercise, and accurately capture the rules of metabolic changes induced by exercise. Artificial intelligence technology was used to analyze the monitoring data to construct an early warning model of metabolic indicators and cardiovascular outcomes, so as to find out the potential risks in the process of exercise intervention in time and dynamically adjust the exercise plan. To explore the remote exercise guidance mode and provide residents with personalized exercise plans, real-time guidance, metabolic index interpretation, and other services through the online platform, so as to improve the accessibility and compliance of exercise intervention.

Although this review strives to comprehensively integrate the available research evidence, there are still some limitations. In human clinical cohort studies, some cohorts suffer from small sample sizes and short follow-up periods, and they primarily focus on specific populations, such as young and middle-aged athletes and diabetic patients at specific stages of the disease. There is a lack of large sample and long-term cohort studies on elderly people and people with multiple organ underlying diseases, which limits the universality of research conclusions. Although animal experiments and *in vitro* studies can accurately reveal the molecular mechanism, there are still some problems, such as species differences and inconsistency between the cellular microenvironment and the physiological environment *in vivo*. The effectiveness of the translation of research results to human clinical needs to be further verified. At present, studies on the dose-effect of exercise intensity, duration, and type are relatively scattered, and the optimal exercise intervention parameters for people with different metabolic states and different CVD risks are not clear. The deep molecular mechanisms of the cross-regulation of intestinal microecology and metabolic pathways by exercise (such as the interaction between fat metabolism and intestinal flora mediated by circadian rhythm) are not well studied. However, there is still a lack of multicenter, prospective intervention studies to confirm the causal relationship between exercise-induced dynamic changes in metabolic indicators and cardiovascular outcomes. In addition, existing studies have not paid enough attention to the synergistic regulation between different metabolic pathways, and it is difficult to comprehensively explain the overall metabolic network mechanism of exercise in protecting cardiovascular health.
